# The incidence of MRSA infections in the United States: is a more comprehensive tracking system needed?

**DOI:** 10.1186/s13756-017-0193-0

**Published:** 2017-04-07

**Authors:** Kevin T. Kavanagh, Said Abusalem, Lindsay E. Calderon

**Affiliations:** 1 Health Watch, Lexington, KY USA; 2 Health Watch, University of Louisville, Louisville, KY USA; 3 Health Watch, Eastern Kentucky University, Richmond, KY USA

**Keywords:** Surveillance, MRSA, Methicillin-resistant Staphylococcus aureus, EIP, Emerging Infection Program, Multi-drug resistant organisms, Epidemiology, MDRO, VA, UHC

## Abstract

A review of epidemiological studies on the incidence of MRSA infections overtime was performed along with an analysis of data available for download from Hospital Compare (https://data.medicare.gov/data/hospital-compare). We found the estimations of the incidence of MRSA infections varied widely depending upon the type of population studied, the types of infections captured and in the definitions and terminology used to describe the results. We could not find definitive evidence that the incidence of MRSA infections in U.S. community or facilities is decreasing significantly. Of concern are recent data reported to the National Healthcare Safety Network (NHSN) on MRSA bloodstream infections which indicate that by the end of 2015 there had been little change in the average facility Standardized Infection Ratio (0.988), compared to a 2010–2011 baseline and is significantly increased compared to the previous year. This is in contradistinction to the recent Veterans Administration study which reported over an 80% reduction in MRSA infections. However, this discrepancy may be due to the inability to reconcile the baselines of the two data sets; and the observed increase may be artifactual due to aberrations in the NHSN tracking system. Our review supports the need for implementation of a comprehensive tracking and monitoring system involving all types of healthcare facilities for multi-drug resistant organisms, along with concomitant funding for both staff and infrastructure. Without such a system, determining the effectiveness of interventions such as antibiotic stewardship and chlorhexidine bathing will be hindered.

## Introduction

Recent investigative reports in the media has brought into question the adequacy of the United States’ Methicillin-resistant *Staphylococcus aureus* (MRSA) tracking system along with whether the epidemic of MRSA is being brought under control [1].

Currently the United States has adopted a “one size does not fit all” approach and has relatively few mandates related to infection control. The Presidential Advisory Council on Combating Antibiotic-Resistant Bacteria is studying these issues, but data supporting the contention that the MRSA epidemic is being brought under control appears to be largely derived from the Emerging Infection Program (EIP). The data from this program has come under criticism by the news media for its sample size, age and limited geographic representation [1].

Having a standardized reportable methodology with comparative data is critical to enable both researchers and policy makers to formulate and implement effective protocols to confront the epidemic of MRSA in the United States. The following are major epidemiology reports with varying methods of tracking and reporting of MRSA infections.

### Emerging infection program (EIP)

**Fig. 1:**
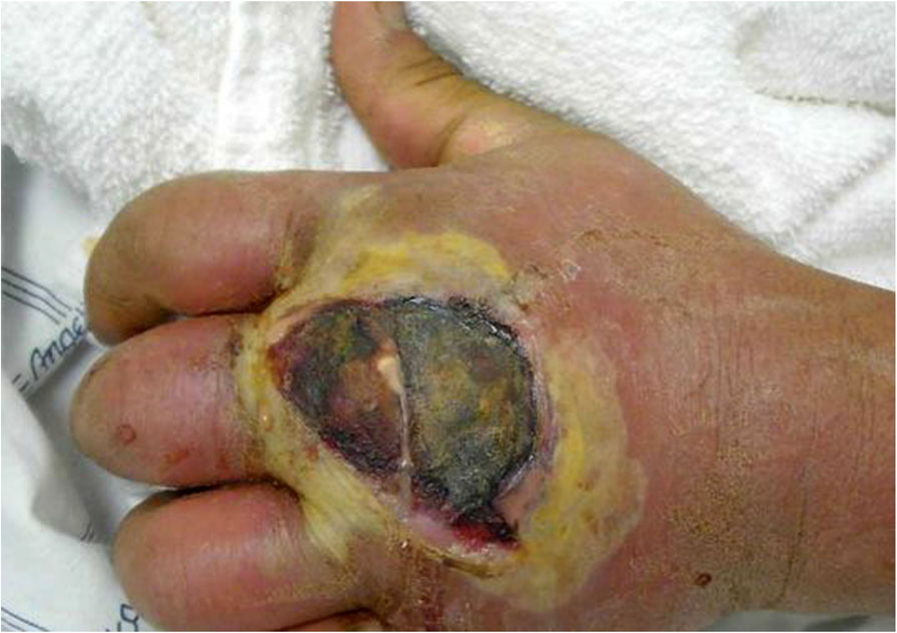
MRSA infection which would not necessarily be reportable under the bloodstream or invasive infection metrics. Centers of Disease Control and Prevention photo archive (Photo credit: Gregory Moran, M.D)

There are three major papers which summarized progressive results in adult patients from the EIP surveillance program which derives its data from nine metropolitan areas in the United States [2–4]. Since each paper encompasses and adds onto the data previously reported, we will focus our comments on the last report of Dantes, et al. [4]. These studies reported “invasive” MRSA infections as detected by laboratory-based case finding. An invasive infection was defined as a positive MRSA culture from a normally sterile site, such as blood, pleural fluid, peritoneal fluid, cerebral spinal fluid, or bone [2, 4, 5]. Eighty percent of invasive infections had positive blood cultures and only 22% were skin infections [4]. The overall in-hospital fatality rate was 13%. This definition mainly captures severe infections. The infection shown in Fig. 1 would not necessarily be reportable under the invasive infection metric. For data acquisition dates 2005–2011, Dantes, et al., reported a 27.7% decrease for healthcare-associated community-onset, 54.2% decrease in hospital-onset (culture taken greater than three days after admission), but only a 5.0% decrease in community associated infections.

Covering a similar time-period (2005–2010) and using data from the EIP, Iwamoto, et al. [5] reported that in pediatric patients 90 days and older, there was not a significant decrease in hospital-onset, or healthcare-associated community-onset “invasive” MRSA infections. They also observed a 10.2% per year increase in community-associated “invasive” infections. However, they did observe significant decrease in MRSA infections of 11.3% per year in children 3–89 days of age [5].

### The surveillance network (TSN) database-USA

This tracking system contains electronically submitted data from more than 300 laboratories across the United States [6]. For data acquisition years 2005–2008, Klein, et al. [6] reported a stable rate of MRSA related hospitalizations for pneumonia and blood infections; whereas the proportion of cultures with the assumed hospital associated MRSA phenotypes increased. This data appears to contradict the EIP data reported by Kallen, et al. [3] for the same data acquisition dates which showed decreasing MRSA infections, being most pronounced with healthcare-associated infections. Klein, et al. [6] stated a possible explanation for this difference was geographical variability and that their sample had greater geographical representation.

### University healthcare consortium data (UHC)

The UHC estimated MRSA infections per 1000 hospital discharges from Academic Medical Centers and found that MRSA infections, as determined from billing data, doubled (20.9–41.7) between the years of 2003 and 2008 [7].

### National healthcare safety network (NHSN)

The NHSN is a comprehensive reporting system for laboratory identified MRSA bloodstream infections that occur in acute care hospitals which participate with Medicare’s Prospective Payment System. Infections that occur greater than three calendar days after admission are defined as hospital-onset. Infection rates are risk adjusted and compared to a 2010–2011 baseline [8]. However, standardized data are available for MRSA bloodstream infections in less than 2000 of the approximate 5000 acute care facilities in the United States. In addition, small rural hospitals which are defined as critical access and pediatric facilities may not have reported data. Veterans Administration and military hospitals also use a different system for quality control and reporting of infections. Critical access hospitals also tend to have limited resources to confront infection disease and are at risk of disseminating resistant organisms to larger facilities through their referral network.

**Fig. 2:**
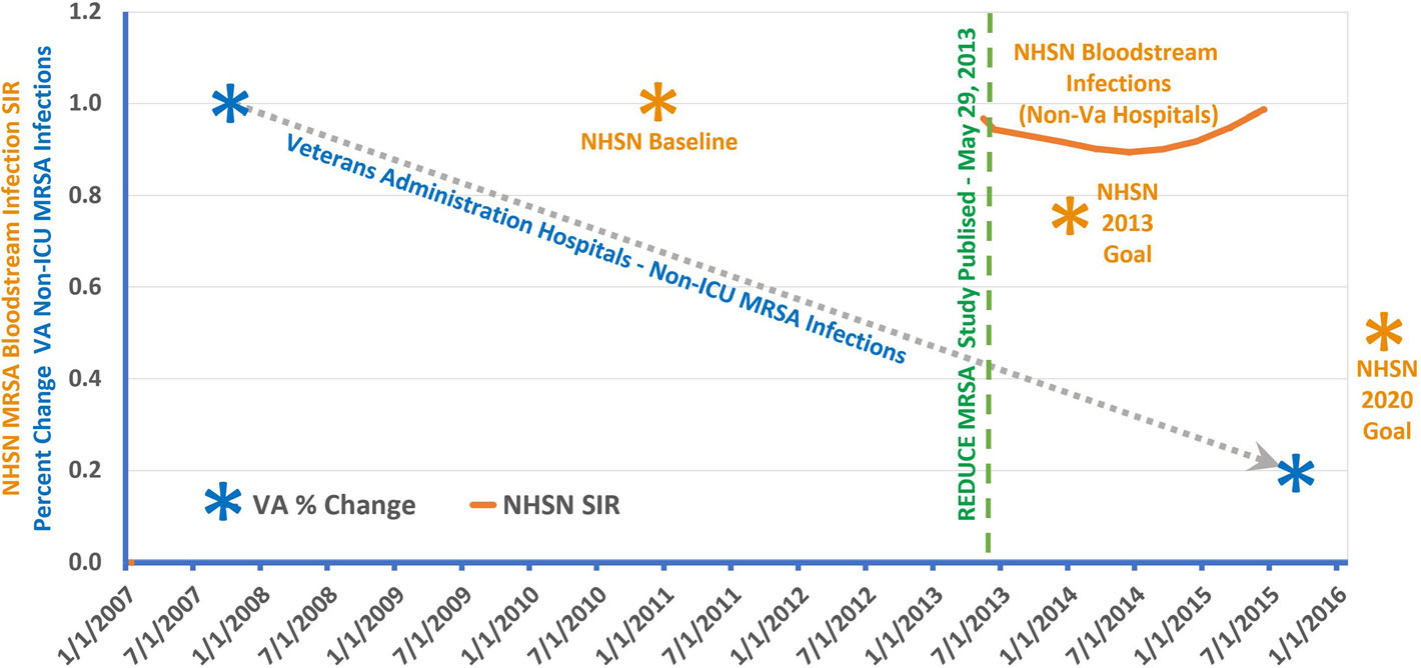
Graph of National (Patient Level) Standardized Infection Ratio (SIR) for MRSA Bloodstream Infections in relationship to MRSA goals and to the performance of VA hospitals in reducing total non-ICU MRSA infections. Data was used only from hospitals that also had a calculable SIR. The data acquisition periods for the SIR are shown in Table 1. Only the baseline and final data points for the VA MRSA Infections are shown

As shown in Fig. 2, there has been little or no change in infection rates compared to the 2010 to 2011 baseline [9]. Initially, there appeared to be a slight decrease, but the rates have trended upward and returned to the baseline. It should be noted that the baseline data was collected under voluntary reporting, as the subsequent data shown in Table 1 was collected with mandatory reporting. Thus, one could argue that the baseline data was from higher performing facilities. In addition, the increase observed in 2015 may have been caused by methodological changes in how community MRSA environmental pressure is calculated. However, the data indicates that there has been little change in hospital-onset MRSA bloodstream infection rates between the initial and final mandatory reporting periods as shown in Fig. 2 and Table 1, and that as of 2015 the United States did not achieve the MRSA bloodstream infection reduction goal of 25% [10].

**Table 1:** Hospital-onset MRSA bloodstream infections – facility level and national (patient level) performance

Acquisition dates	Average facility SIR	Average national SIR	Number of facilities
1/1/2013 to 9/30/2013	0.95876	0.96766	1666
1/1/2013 to 12/31/2013	0.91540	0.94380	1889
7/1/2013 to 6/30/2014	0.91484	0.91766	1906
10/1/2013 to 9/30/2014	0.89426	0.90195	1904
1/1/2014 to 12/31/2014	0.89134	0.89422	1916
4/1/2014 to 3/31/2015	0.89717	0.90124	1911
7/1/2014 to 6/30/2015	0.92568	0.91835	1899
10/1/2014 to 9/30/2015	0.96378	0.94811	1825
1/1/2015 to 12/31/2015	0.98812	0.98740	1830

Data for National Level Performance was derived from hospitals that also had a calculable SIR. NHSN data from https://data.medicare.gov/data/hospital-compare (SIR: Standardized Infection Ratio) (The Facility Level data standard deviation for acquisition dates 1/1/2014 to 12/31/2014 was 0.7730. The Facility Level data standard deviation for acquisition dates 1/1/2015 to 12/31/2015 was 0.8753. Unpaired t-test *P* < 0.0003)

Another observation is that Table 1 is needed to fully explain the data acquisition windows’ length and time periods which are presented in Fig. 2. This adds to the complexity of data analysis and exemplifies the need for a more standardized system of reporting.

### U.S. Military and veterans administration (VA) healthcare systems

U.S. Military facilities and the VA Healthcare Systems have reported dramatic decreases in MRSA infections.

Landrum, et al. [11] reported a decrease in hospital-onset MRSA bacteremia from 0.7 cases per 100,000 person-years to 0.4 cases per 100,000 person-years from 2005 to 2010. Community-onset MRSA bacteremia decreased from 1.7 to 1.2 cases per 100,000 person-years during the same time period. However, there was not a significant decrease in MRSA skin, wound or soft tissue infections for either hospital-onset or community-onset MRSA infections. This report analyzed Tricare beneficiaries treated at U.S. Military facilities and was also based upon laboratory data with hospital-onset infections defined as cultures performed greater than three calendar days after admission.

The VA has also seen a dramatic reduction in MRSA infections in 127 reporting acute care hospitals. From October 2007 to October 2015, healthcare-associated MRSA infection rates dropped 87.0% in ICUs and 80.1% in non-ICU patient areas, achieving an incidence of 0.147 and 0.090 infections per 1,000 patient days, respectively [12]. In long term care facilities the VA reports MRSA infection reductions of 49.4% from July 2009 to September 2015 [12].

Unlike the private sector, government healthcare delivery systems have a standardized delivery system with strong national control. Implementation and adherence to protocols would be expected to be easier. Thus, extrapolating performance data from governmental systems to the private sector should be done with caution.

## Discussion

Five reports which had data acquisition periods which ended on or before 2011 are summarized in Table 2. These reports measured different types of MRSA infections and measured different patient populations (military facilities, medical centers, general population, pediatric patients and those who reside in a restricted geographic area). These variables make comparison of the various study results difficult. Although, many studies reported data showing a decrease in MRSA infections, increases were found in one study for total MRSA infections, in another for community-associated infections and another for the assumed MRSA hospital-associated phenotype. This latter study also observed the rates for MRSA pneumonia and bloodstream infections which remained constant In several of the studies the observed decrease did not reach statistical significance.

**Table 2:** Summary of findings of MRSA epidemiology studies with data acquisition periods ending on or before 2011

		Acquisition Dates	Type of Infection	Patient Population	Geographic Distribution	Results
Emerging Infection Program	Dantes, et al. [4]	2005 to 2011	Invasive MRSA	All Ages: Hospital-Onset	Nine Diverse Metropolitan Areas	54.2% Decrease
	Dantes, et al. [4]	2005 to 2011	Invasive MRSA	All Ages: healthcare- Associated Community-Onset	Nine Diverse Metropolitan Areas	27.7% Decrease
	Dantes, et al. [4]	2005 to 2011	Invasive MRSA	All Ages: Community- Associated	Nine Diverse Metropolitan Areas	5.0% Decrease
	Iwamoto, et al. [5]	2005 to 2010	Invasive MRSA	Pediatric, 90 days and Older: Hospital-Onset	Nine Diverse Metropolitan Areas	8.7% per year Decrease *P* = .10
	Iwamoto, et al. [5]	2005 to 2010	Invasive MRSA	Pediatric, 90 days and Older: Healthcare-Associated Community-Onset	Nine Diverse Metropolitan Areas	2.6% per year Decrease *P* = .60
	Iwamoto, et al. [5]	2005 to 2010	Invasive MRSA	Pediatric, 90 days and Older: Community-Associated	Nine Diverse Metropolitan Areas	10.2% per year Increase *P* = .007
	Iwamoto, et al. [5]	2005 to 2010	Invasive MRSA	Pediatric, 3 to 89 days of age:	Nine Diverse Metropolitan Areas	11.3% per year Decrease
Surveillance Network (TSN) Database-USA	Klein, et al. [6]	2005 to 2008	MRSA Infections from Laboratory isolates combined with NIS Data	MRSA Infections Associated With Being Hospital-Onset	300 Geographic Distributed Laboratories Across The United States	MRSA Pneumonia & Blood Infections Remained Constant, “assumed” hospital-associated MRSA phonotype increased.
University Healthcare Consortium Data	David, et al. [7]	2003 to 2008	MRSA Infections Coded on Admin- istrative (Billing) Data	Patients Discharged From University Hospitals	420 University Hospitals and Affiliated Hospitals	100% Increase
U.S. Military	Landrum, et al. [11]	2005 to 2010	MRSA Bacteremia	Tricare Patients Treated at Military Facilities	Hospital Onset MRSA: Military Personnel, Retirees, Immediate Family treated at 266 Military Facilities	43% Decrease *P* < .005
	Landrum, et al. [11]	2005 to 2010	MRSA Bacteremia	Tricare Patients Treated at Military Facilities	Community Onset: Military Personnel, Retirees, Immediate Family treated at 266 Military Facilities	29% Decrease *P* < .005

Recent data reported from the VA and NHSN are more applicable to policy formulation but both data sets are not comprehensive and are not directly comparable, since both have different baseline dates and one measures total MRSA infections and the other MRSA Bloodstream Infections.

This lack of uniformity of MRSA reporting has hindered the United States’ ability to formulate control strategies. For example, two different categories of control protocols are starting to emerge. One which is similar to the one used by the VA and is based upon identification, isolation and/or decolonization. The other is based on the REDUCE MRSA study [13] and implements a protocol of universal daily bathing with chlorhexidine along with intranasal mupirocin.

As shown in Fig. 2, the VA has reported excellent reductions for MRSA infections compared to little if any reduction that has been observed in recent NHSN data for MRSA bloodstream infections. But how does one compare the baselines? Maybe the reduction in the NHSN data had already occurred? This cannot be determined by the conflicting data in earlier studies (see Table 2). We can only assume that the 2010–2011 baseline shown in Fig. 2 is at an unacceptable level, since the U.S. Department of Health and Human Services has sent a goal of a 50% reduction by 2020 [14].

This is an important question. The protocol for universal daily chlorhexidine bathing which gained rapid popularity in the United States was reported in May 2013 and widely disseminated by Agency of Healthcare Research and Quality in September 2013 [15]. Dr. John Jernigan from the CDC in Jan. 2017 stated that “this practice is now being used routinely in over 60% of hospitals in the United States” [16]. But using the NHSN data (acquisition dates from Jan. 1, 2015 to Dec. 31, 2015) the rates of MRSA bloodstream infections has not appeared to decrease (see Fig. 2) and have now risen almost back to the baseline (Facility SIR = 0.988; *P* < 0.0003 compared to 2014 data with acquisition dates from Jan 1, 2014 to Dec. 31, 2014).

However, even this increase can be questioned due to aberrations in the data caused by compensation for community MRSA environmental pressure on facilities. It should be noted that these types of adjustments are controversial, since they would also be expected to mitigate the impact of facilities not doing surveillance in regions with high environmental MRSA pressure.

Is the United States on the right track and is universal chlorhexidine bathing a policy which should be implemented on a wide spread basis or should the United States be expanding the implementation of MRSA screening and isolation/decolonization protocols? A comprehensive tracking system would be critical in providing data to answer this question.

## Conclusion

With the various study populations and data gathering methodologies, comparisons between epidemiological reports are difficult to make. However, we were not able to identify firm evidence that there has been a significant decrease in total or healthcare-associated MRSA Infections in the United States. In addition, the contradictions between the studies is a testament for the need of a comprehensive tracking system for MRSA and other multi-drug resistant organisms. A comprehensive system should also report infections from all types of healthcare facilities, not just acute care hospitals. With the advent of electronic medical records, the reporting of MRSA cultures and infections along with supplemental information can be automated, making the system less burdensome.

Policymakers in Washington, DC appears to be focused on major funding of the development of new antibiotics. It is evident that one of the first steps we should take is the implementation of a comprehensive tracking system for monitoring resistant organisms, along with concomitant funding for both the staff and infrastructure. Without this, the difficulty achieving the 2020 MRSA reduction goal of 50% will be hindered [14]. Determining the effectiveness of interventions such as antibiotic stewardship and chlorhexidine bathing will be impaired. In addition, the United States may not be able to accurately prioritize antibiotic development, and will have an encumbered early warning system for the emergence of resistance to newly developed antibiotics.

## Abbreviations

EIP: Emerging infection program
MRSA: Methicillin-resistant Staphylococcus aureus
NHSN: National healthcare safety network
NIS: National inpatient sample
SIR: Standardized infection ratio
UHC: University healthcare consortium
VA: Veterans administration
